# Genetics of atrial fibrillation—practical applications for clinical management: if not now, when and how?

**DOI:** 10.1093/cvr/cvab153

**Published:** 2021-05-12

**Authors:** Shinwan Kany, Bruno Reissmann, Andreas Metzner, Paulus Kirchhof, Dawood Darbar, Renate B Schnabel

**Affiliations:** 1 Department of Cardiology, University Heart and Vascular Center, University Medical Center Hamburg Eppendorf, Martinistraße 52, 20251 Hamburg, Hamburg, Germany; 2 German Center for Cardiovascular Research (DZHK), partner site Hamburg/Kiel/Lübeck, Martinistraße 52, 20251 Hamburg, Hamburg, Germany; 3 The Institute of Cardiovascular Sciences, University of Birmingham, Edgbaston Birmingham B15 2TT, UK; 4 Division of Cardiology, Departments of Medicine, University of Illinois at Chicago and Jesse Brown Veterans Administration, 840 South Wood Street, Suite 928 M/C 715, Chicago, IL 60612, USA

**Keywords:** Atrial fibrillation, Antiarrhythmic drugs, Catheter ablation, Response, Arrhythmias, Genetic variation, Genotype

## Abstract

The prevalence and economic burden of atrial fibrillation (AF) are predicted to more than double over the next few decades. In addition to anticoagulation and treatment of concomitant cardiovascular conditions, early and standardized rhythm control therapy reduces cardiovascular outcomes as compared with a rate control approach, favouring the restoration, and maintenance of sinus rhythm safely. Current therapies for rhythm control of AF include antiarrhythmic drugs (AADs) and catheter ablation (CA). However, response in an individual patient is highly variable with some remaining free of AF for long periods on antiarrhythmic therapy, while others require repeat AF ablation within weeks. The limited success of rhythm control therapy for AF is in part related to incomplete understanding of the pathophysiological mechanisms and our inability to predict responses in individual patients. Thus, a major knowledge gap is predicting which patients with AF are likely to respond to rhythm control approach. Over the last decade, tremendous progress has been made in defining the genetic architecture of AF with the identification of rare mutations in cardiac ion channels, signalling molecules, and myocardial structural proteins associated with familial (early-onset) AF. Conversely, genome-wide association studies have identified common variants at over 100 genetic loci and the development of polygenic risk scores has identified high-risk individuals. Although retrospective studies suggest that response to AADs and CA is modulated in part by common genetic variation, the development of a comprehensive clinical and genetic risk score may enable the translation of genetic data to the bedside care of AF patients. Given the economic impact of the AF epidemic, even small changes in therapeutic efficacy may lead to substantial improvements for patients and health care systems.

## Graphical Abstract

**Figure:**
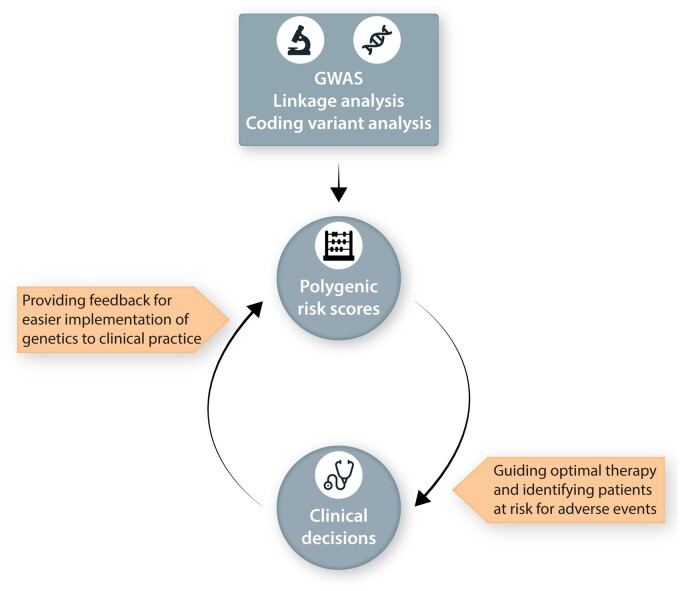

## 1. Introduction

More than 30 million people around the world were affected by atrial fibrillation (AF) in 2010.^1^ The lifetime risk of developing AF in people of European descent older than 40 years one in three and AF is associated with significant morbidity and increased mortality.^2^ Even on optimal current therapy, approximately half of this mortality is cardiovascular, including sudden death and heart failure.^3^ The aging of the population, accumulation of comorbidities associated with AF such as the hypertension, chronic kidney disease, obesity, and heart failure, and improved detection methods will fuel the growing AF epidemic.^4^ The prevalence of AF in the USA (5.2 million cases in 2010) is projected to increase to over 12 million cases by 2030.^5^ Likewise, in whites of European descent the prevalence will rise from 7.6 million cases in 2016 to over 14 million in 2060.^6^

Besides treating the underlying conditions to prevent recurrent AF and reducing AF-related complications, oral anticoagulation can prevent most strokes in patients and rate control therapy reduces AF-associated symptoms. In fact, most patients with AF are managed with these treatment modalities.^7^ However, even on anticoagulation and rate control therapy, and with good management of comorbidities, AF-related complications remain high with 5% annual risk of cardiovascular death, stroke, or hospitalization for worsening of heart failure or acute coronary syndrome.^3^

The current ‘one-size fits all’ approach to selecting rhythm control therapy especially antiarrhythmic drugs (AADs) is based largely on minimizing the risk of adverse effects rather than on therapeutic efficacy.^8^ Possible reasons for the high variability in response to AAD therapy include heterogeneity of the underlying electrical and structural substrate, limited understanding of the underlying pathophysiological mechanisms, and our inability to predict individual responses.^9^ The genomic variability underlying AF also contributes to the variable response, as suggested by recent translational hypothesis-generating studies.^10^ Thus, a major knowledge gap is predicting which patients with AF are most likely to respond to rhythm control therapy. The heritability of AF has been reported as ∼22% in the UK Biobank and as high as ∼62% in a study of Danish twins.^11^^,^^12^

Following the first description of familial AF over seven decades ago,^13^ and almost 15 years after the first publication of a genetic locus associated with AF,^14^ great progress has been made in understanding the genetic architecture of the arrhythmia. Advances in next-generation sequencing have enabled comprehensive genotyping at reasonable cost. Although genetic approaches to AF have revealed that susceptibility to AF development and response to therapy is modulated in part by the underlying genetic substrate, the impact of these discoveries on the bedside care of patients has been limited. In this monograph, we review whether the increased knowledge on common genetic variation associated with AF can improve our management of the arrhythmia and how and when this may be best accomplished.

## 2. Genetic basis of AF

In 1936, Wolff^13^ described a family of three brothers with AF in one of the first reports of a hereditary form of AF. The first discovered gene mutation associated with familial AF was *KCNQ1* coding for the K_v_7.1 potassium channel that mediates the slow rectifying potassium current (I_Ks_).^15^ Other ion channel genes implicated in AF include *SCN5A*, the gene coding the cardiac sodium channel. For instance, in a Japanese family with autosomal dominant familial AF, a novel gain-of-function mutation in *SCN5A* (M1875T) was identified.^16^ Mutations in *SCN5A* have been identified in ∼5% of unselected AF population, with a higher prevalence in younger cohorts.^17^ Single-nucleotide polymorphisms (SNPs) encoding sodium channels (SCN family) have also been associated with AF.^18^ Interestingly, a loss-of-function (LOF) mutation in the potassium voltage-gated channel, shaker-related subfamily member 5 (*KCNA5*) modulated tyrosine kinase regulation which suggests more subtle disruptions to cardiac electrophysiology than just dysfunctional ion channels.^19^

The first and only non-ion channel gene (*NPPA*) linked with familial AF was identified in a large family of 11 affected members.^20^ This frameshift mutation produced a mutant peptide that shortened the action potential duration in an isolated rat heart model and predisposed to re-entrant AF. Importantly, this was the first report that established a link between a non-ion channel mutation and altered atrial electrophysiology. Recently, next-generation sequencing identified two novel variants in myosin heavy chain 6 (*MYH6*) and myosin light chain 4 (*MYL4*) that are associated with familial AF emphasizing the important role of myocardial structural proteins in the pathogenesis of AF.^21^^,^^22^ Mutations of *MYL4* associated with AF and structural cardiac myopathy have been later replicated in humans and animal models including zebrafish and rats.^23^^,^^24^ Of the genes implicated in AF, the strongest level of evidence exists for *MYH6*,^21^  *MYL4*,^22^^,^^23^^,^^25^ titin (*TTN*),^26–28^ and *NPPA*.^20^

Although research into rare variants in affected families has provided important insights into the underlying genetic mechanisms in isolated families, the attributable risk at the population is modest at best.^29^ Traditional linkage analysis and candidate gene approaches are complemented by genome-wide association studies (GWAS). Common variants associated with AF susceptibility have a weaker effect in the individual, but their population-attributable risk is considerable and may enable a genotype-based approach in large clinical trials. The reporting of novel variants in *PLEC* and *TTN* associated with early-onset AF have emphasized the importance of genes encoding for structural proteins in AF pathogenesis. In a GWAS of an Icelandic cohort, a *PLEC* missense variant, which is an important part of the cytoskeleton, was associated with an increased AF risk and cardioembolic stroke.^30^ The effect was, however, weaker compared with variants of *MYH6*.^30^ In a Danish study, titin-truncating variants (*TTNtv*) were associated with familial and early-onset AF with compelling evidence.^26^^,^^31^^,^^32^ Remarkably, 16% of probands with familial AF and 4.7% of patients with early-onset AF carried *TTNtv*. In zebrafish, these variants were associated with a sarcomere defect and atrial fibrosis.^31^ Titin is the largest protein in humans and central to sarcomere integrity by connecting the Z- and M-line. In a case–control study, *TTN* LOF variants were associated with early-onset AF.^26^ Around 2.1% of AF patients carried *TTN* LOF variants compared with 1.1% in control subjects. The prevalence of *TTN* LOF increased with younger age with 6.5% in early-onset AF patients under 30 with the findings replicated in the UK Biobank and the MyCode Geisinger cohort.

Testing for *TTNtv* is increasing in patients with heart failure because of robust data linking these variants with dilated cardiomyopathy (DCM).^33^^,^^34^ Patients with DCM and *TTNtv* have also been reported to be at higher risk for arrhythmias.^35^ Increased surveillance in patients with DCM and *TTNtv* has therefore been proposed to identify patients at risk for worsening heart failure.^36^ However, the benefit of *TTNtv* testing in AF patients without a history of heart failure is remains unclear. In a small case series of 25 patients with early-onset AF (<45 years, without significant comorbidities) referred to a tertiary care centre, 21 had at least one rare variant in a cardiomyopathy-associated gene (4 with *TTN* LOF and 1 with *RMB20* which is implicated in titin splicing).^27^ Importantly, 8 out of 11 patients with structural left ventricular dysfunction by cardiac magnetic resonance imaging were missed by transthoracic echocardiography.^27^ Consequently, there may be a role for sequencing *TTN* in patients with early-onset AF who appear to have normal hearts but may be at risk for developing heart failure. Such an approach may include surveillance for peripartum cardiomyopathy or chemotherapy-induced cardiomyopathy, both of which have been associated with *TTN* variants.^37–39^ Yet, the process of structural or sarcomeric dysfunction in AF is not fully understood. An interesting zebrafish model of *PITX2c* LOF showed early sarcomeric changes in zebrafish embryos before extensive remodelling or electrophysiological defects were detectable.^40^ Metabolic changes with an increase of reactive oxygen species also contributed to sarcomeric changes and arrhythmia development.^40^ Further research will provide us with more insight into the mechanisms of genetic variation leading to AF and actionable findings. One example of that is the association of *KCNN2* and *KCNN3* variants with AF in several GWAS.^41^^,^^42^ Both genes encode proteins of a small conductance calcium-activated potassium channels (K_Ca2_)^43^ that are targeted by a new class of AADs that are currently evaluated in an ongoing Phase 2 trial for conversion of AF to sinus rhythm (NCT04571385). Thus, variants in ion channels, signalling molecules, and myocardial structural proteins have been implicated in AF. Importantly, current antiarrhythmic therapy blocks cardiac ion channels only and a more personalized, i.e., patient-specific, approach needs to target the underlying substrate for AF including signalling molecules and myocardial structural proteins.

Thus far, the strongest data exist for variants on chromosome (chr) 4q25 adjacent to *PITX2* which is known for its important role in pulmonary vein development and cardiogenesis.^44^^,^^45^ In 2007, Gudbjartsson *et al.*^14^ reported that a common variant on chr 4q25 in 35% of a European cohort and in 75% of a Chinese cohort increased the risk of AF by up to 1.72 per copy. Data from human and mouse models indicate high expression of *PITX2* in adults and susceptibility to AF by action potential shortening.^46^^,^^47^ Consistently the chr4q25 locus has been replicated in European cohorts and Asian cohorts^48^ and new AF loci have been identified on chr16q22 (*ZFHX3*) and chr1q21 (*KCNN3*).^41^^,^^49^ Some of the known loci such as chr4q25 were replicated while others such as chr1q21 did not reach statistical significance.^50^^,^^51^ These findings suggest that AF-associated SNPs increase risk of AF and that at least some are likely ethnicity-specific.

Recently, large GWAS studies have reported new loci for common variants with increased AF risk. For instance, a GWAS for AF with ∼1 million subjects reported 163 independent risk variants at 111 genetic loci and 165 candidate AF genes.^52^ These loci include genes that code for structural functionality, embryogenetic cardiac development, intracellular calcium homeostasis, hormone signalling, and ion channels.^52^ However, the strongest association remains with SNPs located on chr4q25 across European, Japanese, and African-American populations.^53^ These findings further emphasize the multifactorial aetiology of AF and highlight the diagnostic difficulty to identify vulnerable populations.

### 2.1 Genetic risk scores

Although genome-wide polygenic risk scores (GPS) are calculated based on a multi-array of variants with the goal of identifying a population at high risk for developing AF, a number of preconditions have to be met. First, the GWAS must be large enough to identify all common variants associated with AF. Second, there must be sufficient power to replicate the GPS in the validation dataset. An early report used 12 variants on chr4q25 to identify patients with a five-fold increased AF risk; an effect observed in European whites and Japanese patients. Lubitz *et al.*^54^ showed that a comprehensive AF GPS was associated with incident AF exceeding associations of clinical risk factors emphasizing the complementary information provided by clinical and genetic factors. A follow-up study using a GPS with up to 719 SNPs reported that persons in the highest quartile were at 67% increased risk of developing AF.^55^ While the authors reported minimal benefit in addition to clinical risk factors, a GPS consisting of 127 variants identified patients with a two-fold increase of odds of cardioembolic stroke. This study illustrated the potential role of GPS for identifying AF populations with suspected cardioembolic stroke. The Stroke Genetics Network showed that a GPS with 934 variants was associated with cardioembolic stroke and stroke of undetermined source but not with large artery atherosclerosis or small artery occlusion.^56^ A GPS with 32 variants in over 50 000 patients from cardiovascular trials showed a four-fold increase in incident stroke in patients with high genetic risk but ‘low-risk’ CHA_2_DS_2_-VASc of 2.^57^ When examining the lifetime risk for AF in patients over 55 years, a GPS with 1000 variants was able to identify patients at high AF risk but low clinical risk.^58^

Recently, an AF GPS with 6.6 million variants in over 500 000 patients identified 6.1% of the general population at three-fold higher risk for AF.^59^ Identifying individuals at three-fold increased risk for developing AF is potentially ‘actionable’ and may lead to enhanced screening and earlier intervention with therapies and preventing transition to persistent or permanent forms of the arrhythmia. Another important report examined how monogenic and polygenic factors influence AF risk and penetrance in the UK Biobank with 0.44% of individuals carrying *TTN* LOF and an AF prevalence of 14% while the top 0.44% of the polygenic risk score subjects had an AF prevalence of 9.3%.^32^ The authors hypothesized that monogenic factors confer high penetrance but the common variants in the GPSGRS explained the broader risk of AF.

This work captures the essence of genetic testing in a clinical setting. While testing for certain variants has a role in specialized settings (e.g. *TTNtv*) because of high penetrance, the utilization in a broad clinical cohort will be dependent on the use of GPS to assess AF risk. Advances in the field will make GPS more feasible in identifying patients at risk in an inpatient and outpatient settings.

### 2.2 Catheter ablation therapy

The identification of ectopic triggers in the pulmonary veins initiating in vulnerable atria (substrate) established catheter ablation (CA) as the first-line strategy for treatment of symptomatic AF.^60^^,^^61^ Notably, CA is more effective than antiarrhythmic therapy for rhythm control therapy, yet maintenance of sinus rhythm often requires repeat ablation procedures.^62^ Despite the general safety of repeat ablation, procedure-related complications occur not infrequently.^63^

The Catheter Ablation vs. Anti-arrhythmic Drug Therapy for Atrial Fibrillation Trial (CABANA) compared CA vs. AADs and reported no significant difference in the primary outcome (composite of death, stroke, bleeding, and cardiac arrest).^64^ However, patients randomized to ablation reported significant improvement in symptomatic burden and quality of life. This finding was corroborated by the randomized Catheter Ablation Compared With Pharmacological Therapy for Atrial Fibrillation (CAPTAF Trial).^65^ While efficacy is high with CA (∼70–75% for paroxysmal AF), response is not uniform and some ∼10–15% of patients have recurrence of AF.^66^ Comorbidities such as obstructive sleep apnoea and obesity are important predictors of AF recurrence but for most patients the reason for recurrence remains unclear.^67^

Electrical reconnection of initially isolated pulmonary veins represents one of the most important drivers for AF recurrence.^68^ However, it remains unclear why some patients have pulmonary vein reconnection and others do not. The recent FIRE and ICE trial showed that cryoballoon is non-inferior to radiofrequency ablation but associated with fewer reconnected pulmonary veins.^62^^,^^68^ Here, genetics may provide important insights into pulmonary vein reconnection especially as rare variants in genes encoding structural cell integrity have been associated with AF.^26^^,^^30^ Importantly, determining which energy source for ablation is likely to have the best outcome for each patient would facilitate a personalized approach to CA of AF. The evaluation of the impact of AF ablation beyond the pulmonary veins, i.e., isolation of the left atrial appendage or the superior vena cava, ablation of complex fractionated electrograms, bidirectional block of linear lesions, and posterior wall ablation continues to evolve.^69^ To date, the clinical benefit of a genotype-based CA approach remains controversial, and additional data are required before definitive recommendations can be made for their use in clinical practice. Due to its importance in cardiogenesis and pulmonary vein formation, common genetic variation at the chr4q25/*PITX2* locus is the most promising to pursue. A recent study showed that low concentrations of left atrial *PITX2* and its surrogate, elevated BMP10 levels, were predictive for AF recurrence after thoracoscopic AF ablation.^10^

The recently reported primary outcome of the EAST-AFNET 4 trial showed that early initiation of rhythm control therapy, using both AAD and AF ablation, can improve outcomes compared to usual care in patients with recently diagnosed AF.^70^ This will increase the clinical use of rhythm control therapy, which thus far has mainly been used in selected patients.^66^ The role of GPS in predicting the population that will benefit the most from rhythm control therapy with better outcomes including less recurrence of AF, reduced strokes and cardiovascular death remains unclear and randomized controlled trials are required. In view of the expected increased use of rhythm control therapy, better ways to deliver effective, accessible therapy to a large population need to be found.

### 2.3 Antiarrhythmic therapy

The most recent ESC guidelines on the management of AF recommend antiarrhythmic therapy for rhythm control in favour of or at least with equal standing with CA in most indications.^61^ However, recommendations on which AAD to use are in part based on adverse events instead of therapeutic efficacy. Flecainide and propafenone have voltage-dependent effects, e.g. maximal effect at high heart rates, and a rapid onset of action after oral intake, which renders them suitable for the ‘pill-in-the-pocket’ approach for symptomatic AF episodes.^71^

Variants of the gene encoding the β1-adrenergic receptor (ADRB1) has been an early focus of a pharmacogenomic approach. In an analysis of 543 patients from the Vanderbilt AF Registry, carriers of the Gly389 genotype responded better to rate control in AF and needed lower doses.^72^ However, in a Japanese cohort of 159 patients with supraventricular arrhythmias, the Gly389 polymorphism decreased antiarrhythmic efficacy of flecainide when co-administered with β-blockers.^73^ Earlier, a substudy of the Beta-Blocker Evaluation of Survival Trial (BEST) investigated the effect of bucindolol on new-onset AF. While 389 Gly carriers showed no difference, in those with 389 arginine homozygotes, bucindolol reduced new-onset AF by 74% (hazard ratio 0.26, 95% confidence interval 0.12–0.57).^74^ This led to the very first trial [GENETIC-AF trial (Phase 2 Genotype-Directed Comparative Effectiveness Trial of Bucindolol and Toprol-XL for the Prevention of Symptomatic Atrial Fibrillation/Atrial Flutter in Patients with Heart Failure)] to determine genetic risk prediction in pharmacotherapy of AF. When 267 heart failure patients carrying the *ADRB1* Arg389Arg genotype were randomized to bucindolol or metoprolol^75^, there was no difference in new-onset AF or atrial flutter. However, the study was small and a larger Phase 2 trial is needed.

Other AADs used for the treatment of AF include amiodarone, dronedarone, and Class III drugs. In Europe, sotalol is often used to maintain sinus rhythm while dofetilide is prescribed frequently in the USA. Importantly, dronedarone and amiodarone affect multiple other channels, including inhibition of the cardiac sodium and potassium channels but also other currents (I_Kr_, I_Ks,_ I_K1,_ I_Na_).^76^ Amiodarone is more potent than other AADs,^77^ and the only agent that can be safely used in patients with AF and heart failure with reduced ejection fraction.^78^ However, amiodarone’s non-cardiac side effects limit its use as a first-line agent in patients in whom other AADs are available. Importantly, amiodarone is not approved by the FDA for AF.^79^ Alternatives include dronedarone or sotalol which have been shown to have less efficacy and in some cases higher rates of QT prolongation and torsades de pointes.^79^ In conclusion, AAD therapy is complex and requires deep understanding of mechanisms of action and the associated physiological consequences. This leads to underutilization and discordance of clinical practice to guideline recommendations.^80^^,^^81^

### 2.4 Clinical predictors of response to AADs for AF

Due to the proarrhythmic effects of AADs in some patients, researchers have long sought predicting response. In 1980, therapeutic efficacy of AADs for ventricular tachycardia was assessed by suppression of pacing-induced arrhythmia during an electrophysiology study.^82^ Drugs that suppressed pacing-induced ventricular tachycardia were more effective in maintaining freedom from arrhythmia.^82^ Shortly after, a randomized trial confirmed the findings.^83^ However, predicting response to AAD treatment in AF is more challenging as this approach cannot be used to assess therapeutic efficacy due to the chaotic nature of AF and unclear mechanisms. Generally, rhythm control therapies are less efficacious in persistent or longstanding persistent forms of AF and in patients with enlarged atria or poorly treated comorbidities such as hypertension.^84^^,^^85^ However, most studies are limited to investigating AF recurrence after CA.

A recent study examined the impact of obesity on response to antiarrhythmic therapy in 311 patients with AF and in diet-induced obese mice.^86^ Obese patients were less likely to respond to sodium channel blocker AADs compared with non-obese controls. Similarly, treating obese mice with sotalol was associated with a greater reduction in pacing-induced AF burden as compared with sodium channel blockers.^86^ The same group showed in a separate study that there was reduced expression of the cardiac sodium channel in obese mice while potassium channel expression and atrial fibrosis were increased, confirming their findings in patients.^87^

### 2.5 Genotype-based approach to the treatment of AF—where are we now? Antiarrhythmic therapy

Variability in response to pharmacological and non-pharmacological therapy is established in cardiovascular medicine. Recognizing that common genetic variants increase susceptibility to AF, raises the possibility that they may also modulate responses to rhythm control therapy. One of the first pharmacogenetic studies investigated whether response to AADs for symptomatic AF was modified by the angiotensin-converting enzyme (*ACE*) *I/D* polymorphism.^88^ This 287 base pair region within intron 16, associated with increased ACE activity and cardiac fibrosis, was a significant predictor of failure to respond to AADs in patients with early-onset AF.^89^ There was a graded response with patients carrying the ACE *II* genotype experiencing the greatest reduction of symptoms while those with the *DD* genotype responded poorly. A second study evaluated if SNPs on chr4q25, chr16q22, and chr1q21 modulated response to AADs for AF. Only the rs10033464 SNP on chr4q25 was an independent predictor of successful rhythm control with patients carrying the ancestral allele having ∼four-fold increased odds of maintaining sinus rhythm.^90^ There was also a differential response to Class I vs. Class III AADs with individuals carrying the rs10033464 variant allele responding better to Class I as compared to Class III. However, both analyses were performed in whites of European descent, and it remains unclear if the same effect is seen across race–ethnicity. While the findings have to be replicated in other, contemporary cohorts, in 2016, Syeda *et al.*^91^ reported that variable *PITX2* expression not only modulated atrial resting membrane potential but also confirmed the clinical observation that flecainide was more effective at suppressing AF than sotalol in *Pitx2c*^+/−^ mice. There is, however, no data on *PITX2* guided antiarrhythmic therapy in humans.

Overall, the observed effects in genetic risk prediction in AAD therapy are modest and with the rising importance of CA, GPS studies on AAD response have not been pursued. As rare variants do not confer a large population-attributable risk, a GPS approach will need to be developed. With an expected rise of rhythm control therapy after EAST, CA centres will not be able to offer enough patients a procedure, consequently many patients will remain on AAD. There is tremendous potential in the application of genetic risk scoring to guide AAD therapy in a broad and diverse population. It is important to emphasize that almost all pharmacogenetics studies evaluating response to AADs for AF have not been replicated and the effects are modest, reinforcing the need for large scale replication and performing randomized clinical trials before such approaches are introduced into clinical practice.

### 2.6 Ablation therapy

**Figure 1:**
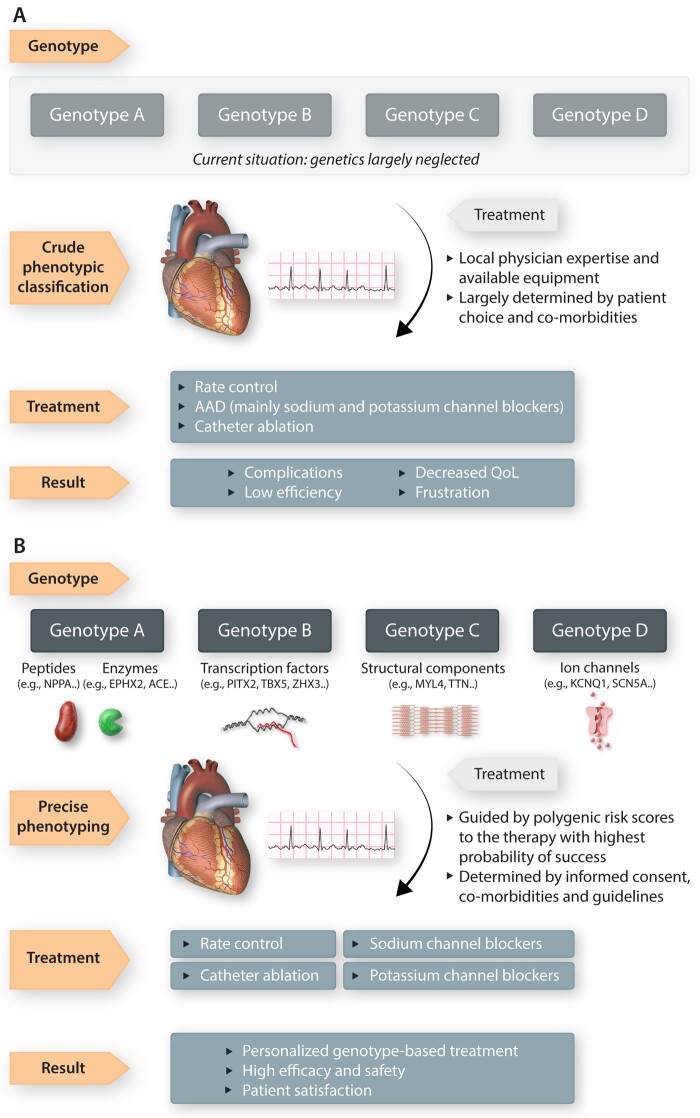
Overview of current (*A*) and proposed (*B*) genotype-guided therapy for atrial fibrillation. AAD, antiarrhythmic drug; QoL, quality of life.

The prediction of AF recurrence after CA may help identify patients who would benefit from frequent follow-up and electrocardiographic surveillance (*Figure 1*). Earlier studies using a candidate gene approach have assessed recurrence of AF after ablation. Similar to risk for new-onset AF, *PITX2* was a major focus for AF recurrence after CA. Two chr4q25 SNPs (rs2200733 and rs10033464; surrogate markers for *PITX2*) were investigated in a German cohort after CA.^92^ While clinical and echocardiographic variables failed to predict recurrence, any variant alleles were associated with early and late recurrence of atrial arrhythmias after CA.

One study examined whether the *ACE I/D* polymorphism predicted recurrence of AF after CA. Confirming earlier findings with antiarrhythmic therapy, the *DD* genotype and left atrial enlargement were significantly associated with AF recurrence.^93^ Importantly, these early studies suggested that genes implicated in the pathogenesis of AF may not only predict AF risk but also response to therapy and should be assessed individually. Epoxyeicosatrienoic acids encoded by the gene *EPHX2* have been implicated as modulators of cardiac ion channels.^94^ A German study with 218 allele carriers and 268 controls showed that the rs751141 variant in *EPHX2* is associated with increased risk of AF recurrence after ablation.^95^ As nitric oxide has been implicated in modulating cardiac vagal activity and cardiac remodelling,^96^ a study of 1000 Korean patients showed that the rs1799983 SNP in the *eNOS3* gene was associated with early recurrence of AF.^97^ Several studies in patients of Asian ancestry showed that inflammatory pathways [interleukin-6 (*ILR6*) gene in a Chinese population],^98^ heme oxygenase-1 gene (*HO-1*) in a Taiwanese cohort,^99^ and angiotensinogen *M235T* polymorphism in a Chinese population though not significant in this cohort, may have value as predictors of AF recurrence after ablation.^100^ In another study, an *SCN10A* SNP (rs6795970) in a Chinese population independently increased the risk of AF recurrence.^101^ However, the value of screening for random rare variants as predictors of AF recurrence after ablation remains questionable. For example, rare variants in cardiac sodium channel genes (*SCN5A, SCN1B–4B)* were not significantly associated with outcome of CA in a Caucasian population.^102^

As earlier studies suggested that rs2200733 and rs10033464 SNPs on chr4q25 were significant predictors of early and late recurrence of AF, Shoemaker *et al.*^103^ performed a GWAS to verify these findings in 991 patients. Only rs2200733 on chr4q25 was significantly associated with recurrence of atrial arrhythmias. In contrast, a Korean study with 1068 AF patients found that no AF genetic loci predicted recurrence after CA.^104^ However, these findings should be viewed in the context of different race–ethnicity studied (European whites vs. Asians). Nonetheless, a recent Korean study showed that a GPS consisting of five SNPs on chr4q25 and one from chr16q22 was moderately predictive of AF recurrence after ablation.^105^ A histidine-rich calcium-binding protein SNP (rs3745297) was associated with a recurrence after CA in a Japanese cohort of 382 patients.^106^

Recently, a large multi-centre study investigated AF recurrence after CA in a large cohort of European ancestry (*n* = 3259) using a comprehensive polygenic risk score consisting of 929 SNPs.^107^ Although there was no significant correlation between the polygenic risk score and AF recurrence, common genetic variation particularly at the chr4q25 locus near *PITX2* was significantly associated with AF recurrence. *PITX2* variants are of clinical and pathophysiological interest, since pulmonary veins are the only established target for CA of AF. While this study suggested that AF recurrence after CA may represent a genetically different phenotype, identifying common genetic variants associated with recurrence is important and is being evaluated in ongoing studies. Their clinical value in predicting response to CA and/or other therapies for AF need to be demonstrated in randomized clinical trials. Many earlier studies using candidate SNPs have not been replicated and robust data are lacking. The value of a candidate SNP-based approach is debatable and questions remain on the number of candidate SNPs were initially investigated in each study. However, the GPS remains the most promising approach for the clinical setting but is not yet ready for prime time. *Table 1* summarizes the existing literature on genomic risk for AF recurrence after CA.

**Table 1:** Overview of studies investigating genome-guided therapy for AF

Gene or SNP	Study design	Primary end point	Results	Statistical value	Reference
ACE I/D polymorphism	Response to AAD therapy (Caucasian, Vanderbilt AF Registry)	Success of therapy defined as ≥75% reduction in symptomatic AF burden (questionnaire based)	ACE DD/ID genotype in early-onset AF a significant predictor of inadequate response to AAD	OR 2.251, 95% CI 1.056–4.798; *P* = 0.036	^88^
Chr4q25: rs2200733, rs10033464 Chr16q22: rs7193343 Chr1q21: rs13376333	Response to AAD therapy (Caucasian, Vanderbilt AF Registry)	Success of therapy defined as ≥75% reduction in symptomatic AF burden (questionnaire based)	SNP rs10033464 on 4q25 predicts AF recurrence	OR 3.27, 95% CI 1.7–6.0; *P* < 0.001	^90^
Variants in 4q25, 16q22, 1q21, β1-adrenergic receptor (*ARDB1*)	Response to AAD therapy in relation to OSA (Caucasian, Vanderbilt AF Registry)	Success of therapy defined as ≥75% reduction in symptomatic AF burden (questionnaire based)	4q25 (rs10033464) locus associated with increase of AAD response in patients with mild OSA	OR 10.0, 95% CI 1.03–97.5; *P* < 0.05	^108^
Recurrence after CA					
Chr4q25 SNPs (rs2200733, rs10033464)	Single-centre study (Caucasian, Leipzig Heart Center AF ablation registry)	Recurrence of AF after CA	rs2200733 and rs10033464 associated with early and late AF recurrence	Early recurrence (OR 1.994, 95% CI 1.036–3.837; *P* = 0.039) Late recurrence (OR 4.182, 95% CI 1.318–12.664; *P* = 0.011)	^92^
*HO-1* gene polymorphism (number of GT repeats)	Single-centre study (Taiwanese-Asian, Veterans General Hospital Taipeh)	Recurrence of AF after CA	<29 GT repeats associated with increased risk of AF recurrence	HR 1.79, 95% CI 1.10–2.94; *P* = 0.02	^99^
Angiotensin-converting enzyme (ACE) I/D polymorphism	Single-centre study (Caucasian, Leipzig Heart Center AF ablation registry)	Recurrence of AF after CA	ACE DD genotype associated with AF recurrence	OR 2.251, 95% CI 1.056–4.798; *P* = 0.036	^93^
*EPHX2* polymorphisms (rs41507953, rs751141)	Single-centre study (Caucasian, Charité Berlin)	Recurrence of AF after CA	rs751141 significantly increases risk of AF recurrence	12 months (OR 3.2, 95% CI 1.237–8.276; *P* = 0.016) 24 months (OR 6.076, 95% CI 2.244–6.451; *P* < 0.0001)	^95^
*IL6R* gene polymorphism (rs4845625)	Retrospective study (Chinese-Han, GeneID population)	Recurrence of AF after CA	rs4845625 increases risk of early and late AF recurrence	OR 1.92, 95% CI 1.30–2.81; *P* = 0.001	^98^
*eNOS3* gene polymorphism (rs1799983)	Single-centre study (Korean-Asian, Yonsei AF Ablation Cohort)	Recurrence of AF after CA	rs1799983 increases risk of early recurrence of AF	OR 1.71, 95% CI 1.06–2.79; *P *=* *0.028	^97^
Chr4q25 (rs2200733, rs10033464), Chr1q21 (rs13376333) Chr 16q22 (rs7193343)	Multi-centre meta-analysis (Caucasian)	Recurrence of AF after CA	4q25 SNP (rs2200733) predicted increase of risk of recurrence	HR 1.3, 95% CI 1.1–1.6; *P* = 0.011	^103^
Chr4q25 (rs6817105, rs2200733), Chr16q22(rs2106261), Chr1q21(rs13376333)	Single-centre study (Korean-Asian, Yonsei AF Ablation Cohort)	Recurrence of AF after CA	No significant correlation between SNPs and outcome	1068 AF patients and 1068 age- and sex-matched controls	^104^
*M235T* polymorphism	Single-centre study (Chinese-Han, Shanghai Jiao Tong University)	Recurrence of AF after CA	M235T variants were not significantly associated with AF recurrence	150 symptomatic drug-refractory AF patients	^100^
*SCN5A, SCN1B–4B, CAV3*, GPD1L, *SNTA1* and *MOG1* variants	Single-centre study (Caucasian, Leipzig Heart Center AF ablation registry)	Recurrence of AF after CA	No association between rare variants and ablation outcome	137 patients	^102^
*SCN10A* SNP (rs6800541)	Single-centre study (Chinese-Han, Shanghai Jiao Tong University)	Recurrence of AF after CA	rs6800541 is and independent risk factor for AF recurrence	OR 0.36, 95% CI 0.22–0.60; *P* = 7.04 × 10^−5^	^101^
The HRC SNP, rs3745297 (T > G, Ser96Ala)	Single-centre (Japanese, Hiroshima University Hospital)	Recurrence of AF after CA	rs3745297 associated with increased risk of AF recurrence	HR 2.66, CI 1.32–5.0; *P* = 0.007	^106^
Polygenic risk score of 5 SNPs [rs1448818, rs2200733, rs6843082, rs6838973 at Chr4q25 (*PITX2*) and 1 SNP rs2106261 at Chr 16q22 (*ZFHX3*)]	Two-centre study (Korean)	Recurrence of AF after CA	Association of recurrence risk and risk score at intermediate or high risk	Intermediate risk (HR 2.00, 95% CI 0.99–4.04) and high risk (HR 2.66, 95% CI 1.32–5.37)	^105^
Polygenic risk score of 929 SNPs	Multi-centre study (Caucasian cohort)	Recurrence of AF after CA	No association of polygenic risk score and AF recurrence	3259 patients from 10 centres	^107^

AAD, antiarrhythmic drugs; AF, atrial fibrillation; CA, catheter ablation; Chr, chromosome; CI, confidence interval; HR, hazard ratio; OR, odds ratio; OSA, obstructive sleep apnoea; SNP, single-nucleotide polymorphism.

## 3. Current value of genetics in clinical application

Over the last decade, tremendous progress has been made in defining the genetic architecture of AF with the identification of rare and common genetic variants. However, there is little evidence that existing data from genetic studies are used for clinical decision-making in AF patients in part because of poor understanding of the underlying genetic substrate for AF, the lack of prospective randomized trials, moderate effect sizes, and variability across race and ethnicity. Larger datasets and ongoing biobanking of samples from large cohorts and rhythm control studies may provide deeper genetic insights and guide selection of antiarrhythmic therapy. Although retrospective studies suggest that response to AADs and CA is modulated by common genetic variation, the development of a comprehensive clinical and genetic risk score may enable the translation of genetic discoveries to the bedside care of AF patients.

Polygenic risk scores may be useful tools to identify patients with good therapeutic response or upfront exclude patients from rhythm control therapies that may be associated with proarrhythmia or non-cardiac toxicities. However, the clinical use of a genetic risk score may be limited to the race–ethnicity of the initial GWAS. Many AF studies were performed with data from the UK Biobank, which is not representative of the general population as often indicated. Participants are healthier, more likely to be female and from less socioeconomically deprived locations.^109^ Studies suggest that not only does the genetic risk of AF vary by race–ethnicity but also response to rhythm control therapy (see *Table 1*). Since most genetic studies have been performed in whites of European descent, the risk of increasing health disparities is apparent and should be carefully considered. Greater diversification of GWAS may alleviate a bias towards white patients of European descent.^110^ Importantly, the translation of genetic data to the bedside is also hindered by technological limitations. GWAS and GPS remain a scientific approach that is available in only advanced cardiovascular research centres. Rapid genetic testing or point-of-care analysis to calculate the GPS are future tools that may enable the comprehensive use of AF genetic data in a clinical setting. The tools must be cost-effective to ensure they are available to all patients and not only benefit those who can afford it. The derivation of GPS for AF recurrence from GWAS of new-onset AF risk (e.g. two different clinical questions) may also be a hindrance to clinical application. The screening for undiagnosed AF achievable with far cheaper competing methods^111^ and clinical risk prediction using novel methods such as machine learning are being evaluated.^112^ Currently, novel loci for AF risk are valuable for understanding the complex pathophysiology of AF but are of limited use for a clinician to guide his therapy. The risk prediction for stroke of unknown source due to undetected AF would have immediate clinical impact, e.g. anticoagulation, if validated in a prospective and randomized clinical trial.

## 4. Future perspective on clinical applications of AF genetics

Currently, the major limitation to translation of genetic data to the care of AF patients is the lack of prospective, adequately powered randomized clinical trials demonstrating improved outcomes. Most studies on AF genetics focus on ‘lone AF’ (older studies) and ‘early-onset AF’ (recent studies) which are both loosely defined and rarely used at the bedside. The latter for instance is not mentioned in the most recent ESC AF guidelines.^61^ Future studies on genetic AF risk and outcomes after therapy must use the clinical terminology of paroxysmal AF, persistent AF, and longstanding persistent AF especially since most guideline recommendations are based on this classification. The often used binary classification of AF or no AF will be less important in the future with AF burden becoming the metric of choice to assess therapeutic efficacy.^113^ An ongoing cross-over study is investigating whether common genetic variants associated with AF modulate response to Class I vs. Class III AADs in patients with symptomatic AF (NCT02347111). Genetic sub-analysis of EAST-AFNET 4 and CABANA trials will also provide new perspectives for genetics-based targeted therapies for AF. The insights from these trials will not only provide new data to improve targeted genetic testing but also help develop a comprehensive clinical and genetic risk score enabling the translation of genetic data to the bedside care of patients with AF. All the major cardiovascular trials must include a genetic substudy to build genetic data on those that are treated (sicker, inpatient cohorts) rather than healthier biobank cohorts. This would also provide clinical and randomized data for the application of GPS in many trial populations whose results often shape guidelines.

Steps are needed to bring AF genetics from bench to bedside including:

1. Increase genetic data on AF patients and those with concomitant disease.
2. Accompany major cardiovascular trials with a genetic substudy.
3. Use polygenic risk scores to predict the probability of a disease-related event.
4. Derive polygenic risk scores for a specific prediction from GWAS data of the relevant cohort.
5. Integrate clinical and genetic risk data into a comprehensive risk prediction tool.
6. Use the clinically relevant classification of AF (paroxysmal, persistent, longstanding persistent).
7. Binary AF constellation will lose importance, use AF burden (via implanted loop recorder).
8. Be aware of racial and socio-economic disparities that GPS usage will increase.
9. Make genotyping cheaper and accessible to non-academic centres

Considering the economic burden and number of patients with AF by 2030 (∼12 million), even small improvements in personalized care by the identification of individuals who benefit the most from antiarrhythmic therapy or CA could lead to substantial improvements in patient outcomes and reduction in health care costs.

## Funding

D.D. is supported by the National Institutes of Health (NIH) grants R01 HL138737, T32 HL139439, T32 HL139439, and 1R01 HL150586. R.B.S. has received funding from the European Research Council (ERC) under the European Union’s Horizon 2020 research and innovation program under the grant agreement No. 648131, from the European Union’s Horizon 2020 research and innovation program under the grant agreement No. 847770 (AFFECT-EU), and German Center for Cardiovascular Research (DZHK e.V.) (81Z1710103); German Ministry of Research and Education (BMBF 01ZX1408A) and ERACoSysMed3 (031L0239). P.K. was partially supported by European Union BigData@Heart (grant agreement EU IMI 116074), British Heart Foundation (FS/13/43/30324; PG/17/30/32961; and PG/20/22/35093), German Centre for Cardiovascular Research supported by the German Ministry of Education and Research (DZHK, via a grant to AFNET), and Leducq Foundation. P.K. is listed as inventor on two patents held by University of Birmingham (Atrial Fibrillation Therapy WO 2015140571, Markers for Atrial Fibrillation WO 2016012783). P.K. receives research support for basic, translational, and clinical research projects from European Union, British Heart Foundation, Leducq Foundation, Medical Research Council (UK), and German Centre for Cardiovascular Research.

**Conflict of interest:** R.B.S. has received lecture fees and advisory board fees from BMS/Pfizer outside this work. P.K. receives research support for basic, translational, and clinical research projects from European Union, British Heart Foundation, Leducq Foundation, Medical Research Council (UK), and German Centre for Cardiovascular Research, from several drug and device companies active in atrial fibrillation and has received honoraria from several such companies in the past, but not in the last 3 years. All other authors declared no conflict of interest.

## Notes

This article is part of the Spotlight Issue on Atrial Fibrillation.
